# Predictive Value of Left Atrial Strain for Atrial High‐Rate Episodes in Patients With Permanent Cardiac Pacing

**DOI:** 10.1111/jce.70186

**Published:** 2025-11-18

**Authors:** Gabriele Dell'Era, Domenico D'Amario, Leonardo Grisafi, Anna Degiovanni, Anthea Tonia D'Amico, Maria Gabriella Guidetti, Luca Pescarmona, Marco Mennuni, Giuseppe Patti

**Affiliations:** ^1^ Maggiore Della Carità Hospital Novara Italy; ^2^ Department of Translational Medicine University of Eastern Piedmont Novara Italy

**Keywords:** atrial fibrillation (AF), atrial high rate episodes (AHRE), cardiac implantable electronic devices (CIED), cardiac pacemakers (PM), left atrial strain (LAS)

## Abstract

**Background:**

Atrial high‐rate episodes (AHREs) detected by cardiac implantable electronic devices are associated with a higher risk of developing subsequent atrial fibrillation. Thus, risk stratification for AHREs is essential to guide patient management and improve outcome. Left atrial strain (LAS) parameters have been proposed as markers of atrial mechanical dysfunction, but their predictive value for AHRE prediction is not definitely established.

**Methods:**

We performed a retrospective, single‐center study on consecutive patients undergoing pacemaker implantation. Pre‐implant echocardiographic assessment included the measurement of LAS parameters. AHREs were defined as asymptomatic episodes with atrial rate > 175 bpm lasting ≥ 5 min by pacemaker evaluation. Primary endpoint was the predictive accuracy of LAS values for AHRE occurrence during follow‐up.

**Results:**

A total of 269 patients (age 78.0 ± 11.2 years; CHA₂DS₂‐VASc score 3.8 ± 1.6) were included. Median follow‐up was 28 months. ROC analysis showed that LAS contraction had superior predictive value for AHREs than LAS reservoir (AUC 0.75 vs. 0.63, *p* = 0.001). A 10.2% cutoff of LAS contraction had sensitivity of 77%, specificity of 64%, and negative predictive value (NPV) of 90% for AHRE occurrence. Kaplan–Meier analysis showed a higher AHRE incidence in patients with LAS contraction < 10.2% (45% vs. 10% in those with LAS contraction ≥ 10.2%; log‐rank *p* < 0.001). Multivariate analysis confirmed LAS contraction < 10.2% as an independent predictor of AHREs (adjusted hazard ratio [aHR] 5.0 (95% CI 2.7–9.3); *p* < 0.001). The risk increase was even higher when a LAS contraction < 10.2% was associated with age ≥ 70 years (aHR 16.3; 95% CI 2.0–131.7; *p* = 0.009).

**Conclusions:**

In patients with a permanent pacemaker, pre‐implant LAS contraction by echocardiography is an independent predictor of future AHRE development, with a very high NPV. The evaluation of LAS contraction may represent a valuable tool for identifying in clinical practice those individuals at low risk for AHREs.

## 1 Introduction

Atrial high‐rate episodes (AHREs) are atrial tachyarrhythmias detected by cardiac implantable electronic devices (CIEDs). The 2017 European Heart Rhythm Association (EHRA) consensus defined AHRE as atrial tachyarrhythmias > 190 bpm detected by CIEDs [1]. The most recent 2024 European Society of Cardiology guidelines on atrial fibrillation (AF) have refined this definition including asymptomatic atrial episodes with a rate > 175 bpm and duration ≥ 5 min in patients without a prior clinical history of AF [2]. These episodes are frequently observed in clinical practice, occurring in over one‐third of patients with CIEDs during a follow‐up period of 2.4 ± 1.7 years [3]. Although initially considered benign, a growing evidence supported that AHREs are associated with a two‐ to fivefold increased risk of clinical AF and related complications, including stroke, systemic embolism, heart failure, and cardiovascular mortality [4, 5, 6].

Based on the above, an early identification of individuals at high risk for AHREs is of paramount importance [2]. In fact, an improved risk stratification may better address tailored follow‐up approaches, facilitate an early diagnosis of clinical AF, and guide the implementation of appropriate therapeutic interventions, such as anticoagulation or rhythm control strategies [7]. Furthermore, understanding the likelihood of AHRE development before CIED implantation may influence device selection, particularly in patients with sinus node dysfunction, where certain devices and algorithms have been shown to prevent AF progression [8, 9, 10, 11] and may provide a timely diagnosis by telemetry or remote monitoring. Nonetheless, while CIEDs are highly effective in managing brady‐arrhythmias, they may inadvertently promote AF by inducing atrioventricular dissynchrony or through long‐term ventricular pacing, potentially resulting in pacing‐induced cardiomyopathy [12, 13]. Therefore, both optimized device programming and patient selection are critical for minimizing the risk of atrial arrhythmogenesis.

Various predictors of AHREs and AF have been proposed, mainly represented by clinical parameters, such as age, arterial hypertension, and left atrium (LA) enlargement [14]. More recently, left atrial strain (LAS), derived from two‐dimensional speckle‐tracking echocardiography, has emerged as a promising biomarker of atrial mechanical dysfunction and structural remodeling [15]. Strain is the mechanical expression of deformation of a body, generally expressed as (final dimension‐initial dimension)/initial dimension. It is dimensionless and is usually expressed as a decimal fraction or a percentage. It can be applied to linear, 2‐dimensional and 3‐dimensional measures. LAS quantifies LA phasic function across the reservoir, conduit and contraction phases, each of which plays an important role in left ventricular filling. LAS is provided as a semi‐automated measure by most of the current echocardiographic softwares; reference values and ways to practically assess LAS were provided by Singh et al. from a wide population of 1766 normal subjects [16]. No unanimous consensus on normal LAS values had been reached to date, even if a mean value of 15%–17% is generally accepted for LAS contraction. A reduced LAS—particularly in the reservoir and contraction phases—has been associated with ventricular diastolic dysfunction and a higher risk of developing AF [17, 18, 19]. In particular, a recent study by Wang et al. on a small population demonstrated that reduced LAS during the contractile phase may independently predict the development of AHREs in CIED recipients [20]. Notably, no univocal data on normal and pathologic LAS values are available. Aim of this study was to assess in a larger cohort the prognostic value of pre‐implant LAS and its phasic components in predicting AHRE development, enhancing risk stratification and potentially guiding early intervention strategies in a specific population of patients undergoing pacemaker (PM) implantation.

## 2 Methods

This retrospective, single‐centre study was conducted at the Division of Cardiology, Maggiore della Carità Hospital, Novara, Italy, between January 2021 and December 2023. We included all consecutive patients who underwent PM implantation with atrial sensing for standard bradycardia indications (e.g., sinus node dysfunction, persistent or paroxysmal atrioventricular block, syncope in suspected bradycardia‐bifascicular block, reflex syncope). The minimum follow‐up period to assess clinical outcomes was 1 year, in particular with regard to development of AHREs and/or AF. Exclusion criteria were: (a) pre‐existing atrial arrhythmias, that is, paroxysmal, persistent or permanent AF, atrial tachycardia, or typical/atypical atrial flutter diagnosed before device implantation; AF at the time of implantation; (b) severe heart valve disease or structural heart disease, such as presence of congenital heart disease or prior surgical intervention on the LA, for example, mitral valve repair or replacement; (c) incomplete or poor‐quality images at baseline transthoracic echocardiography, considered unfeasible for speckle tracking analysis (due to poor acoustic window with image quality insufficient or incomplete/inadequate tracking of LA borders); (d) inadequate follow‐up (< 12 months) after PM implantation.

All patients' electronic records were evaluated in search of demographic characteristics, medical history, cardiovascular risk factors, comorbidities, medications, and indication to PM implantation. Pre‐implant transthoracic echocardiography was always performed using high‐quality ultrasound machines (EpiQ, Philips Healthcare, Netherlands; Vivid E9, GE Medical Systems, Horten, Norway), and re‐assessed for the purpose of this study. Speckle‐tracking analysis on pre‐implant echocardiography was conducted using two different semiautomated 2D‐strain software programs. Specifically, 91.8% of patients were analyzed using Compacs Rev 10.10.21 on the EpiQ system (Philips Healthcare, Andover, Massachusetts, USA), and 8.2% using EchoPac on the Vivid E9 system (GE, Milwaukee, Wisconsin, USA). Key echocardiographic parameters had to include LAS measurement, assessed in three components: reservoir strain (LASr), reflecting atrial filling during left ventricular systole; conduit strain (LAScd), reflecting passive emptying of the left atrium; contraction strain (LASct), reflecting active atrial contraction. Additionally, left atrial volume index (LAVi), left ventricular ejection fraction (LVEF), left ventricular end‐diastolic volume (LVEDV), left ventricular end‐systolic volume (LVESV), E/A ratio, E/e' ratio, systolic pulmonary artery pressure (sPAP) and tricuspid annular plane systolic excursion (TAPSE) were recorded [21]. Speckle‐tracking strain analysis was performed on a good quality grayscale apical four‐chamber view loop, acquired with simultaneous electrocardiographic recording, by two experienced cardiologists blinded to the patients' basic echocardiographic measures, clinical history and subsequent occurrence of AHREs, employing the previously described semi‐automated tools and technique [16]: LA endocardium was manually traced and thus the software automatically tracked the region of interest on the LA wall, generating a LAS strain curve cycle with a configuration by ECG R‐R gating beginning with the onset of systole. We then defined the following three components of LA function: LA reservoir (LASr), conduit (LAScd), and contractile or pump (LASct) strains. Measurements were obtained from three consecutive cardiac cycles, and the mean values were used for analysis to minimize variability. Particular attention was paid to achieve optimal image quality and consistent border tracking during the analysis, avoiding manual corrections if the output was consistent to avoid potential biases.

All patients were followed post‐PM implantation by in‐office visits (at least two in the first 12 months, then at least one visit per year), as per standard clinical practice. During follow‐up visits, performed by a dedicated nurse and expert physician, all electrical measures and arrhythmic episodes were recorded, and endocavitary electrograms (EGM) of arrhythmic episodes were stored for revision. When available, remote‐monitoring data from scheduled and unscheduled transmission were reviewed in search of arrhythmias and AHREs. For the purpose of our study, AHREs were defined according to the previously reported 2024 ESC guidelines on Atrial Fibrillation [2], with the aim to identify clinically significant episodes while excluding transient or nonrelevant events, by the evaluation of EGMs to exclude false detections caused by oversensing, lead noise, or non‐physiological signals. All EGMs were assessed by the physician performing in‐office or remote device interrogation, and then reassessed by one of the investigators (A.D.); in the case of discrepancies, the protocol considered the final adjudication being performed by a senior electrophysiologist (G.D.).

Primary endpoint was to assess the predictive value of pre‐implant LAS parameters for the occurrence of AHREs during post‐implant follow‐up. In particular, the study aimed at determining whether reduced LAS values, measured through speckle‐tracking echocardiography before PM implantation, were independent predictors of future AHREs.

### 2.1 Statistical Analysis

Data are presented as mean ± standard deviation (SD) for normally distributed continuous variables, median [interquartile range, IQR] for non‐normally distributed continuous variables, and as counts (percentages) for categorical variables. Normality was assessed using the Shapiro–Wilk test. Comparisons between groups were performed using the Student *t*‐test for normally distributed continuous variables, the Mann–Whitney *U* test for non‐normally distributed continuous variables, and the *χ* ^2^ test for categorical variables. Receiver operating characteristic (ROC) curves were employed to evaluate the predictive performance of LAS parameters for AHREs. Optimal cutoff values for these LAS variables were derived using the Youden index, and corresponding sensitivity, specificity, positive predictive value (PPV), and negative predictive value (NPV) were calculated. Variables were then dichotomized according to these optimal cutoff thresholds. The occurrence of AHREs by LAS parameters strata was assessed using the Kaplan–Meier survival analysis, with differences between groups compared by the Log‐Rank test. A Cox proportional hazards model was subsequently utilized to identify independent predictors of AHREs, with results expressed as Hazard Ratios (HR) and 95% confidence intervals (CI). Variables with a *p*‐value < 0.10 at univariate analysis were included in the multivariate model. Additionally, collinearity among variables was examined via linear regression analysis, and only those with a variance inflation factor (VIF) between 1 and 3 were retained in the final model. All statistical analyses were performed using STATA 18.0 (StataCorp LP, College Station, TX), with a two‐sided *p*‐value < 0.05 considered indicative of statistical significance.

## 3 Results

**Figure 1:**
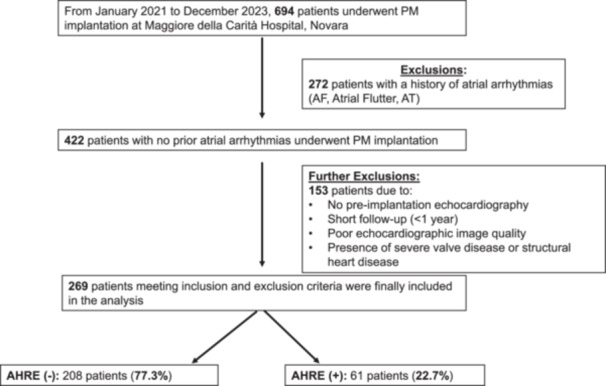
Study design. AF = atrial fibrillation, AHRE = atrial high‐rate episode, AT = atrial tachycardia, PM = pacemaker.

A total of 694 consecutive patients undergoing PM implantation were initially screened, 422 of whom met the eligibility criterion of no prior history of atrial arrhythmias. A total of 153 patients were further excluded due to the absence of pre‐implantation echocardiography, follow‐up < 1 year, poor quality of echocardiographic images or presence of severe valve disease or structural heart disease. Thus, a final cohort of 269 patients represented the study population (Figure 1).

Baseline characteristics of the overall population are reported in Table 1. Mean age was 78.0 ± 11.2 years and prevalence of female gender was 30.9%. Mean CHA₂DS₂‐VASc score was 3.8 ± 1.6. The leading indication for pacing was second‐ or third‐degree atrioventricular block (AVB, 62.8%), followed by sinus node dysfunction (20.1%) (Table S1). The majority of patients received dual‐chamber pacing (DDD mode, 81.0%), predominantly with ventricular leads positioned in the septal or apical regions (56.5%, with the remaining 43.5% receiving conduction system pacing—CSP). Mild‐to‐moderate mitral regurgitation was present in 17.8% of patients, aortic valve stenosis in 6.7% and tricuspid regurgitation in 13.4%. LVEF was 57.0% [95% CI 53.0%–61.0%]. LAS parameters included a reservoir value of 23.4% [16.4–32.4], a contraction value of 10.7% [6.6–17.4] and a conduit value of 11.4% [6.7–17.3] (Table S2).

**Table 1:** Baseline characteristics.

	Overall population (*N* = 269)
Demographic and anthropometric characteristics	
Age (years)	78.0 ± 11.2
BMI (Kg/m²)	25.7 [23.4–28.7]
Female gender	83 (30.9)
Cardiovascular risk factors and comorbidities	
Systemic hypertension	208 (77.3)
Diabetes mellitus	78 (29.0)
Smoking history	102 (37.9)
CKD	54 (20.1)
Dysthyroidism	31 (11.5)
COPD	21 (7.8)
History of stroke/TIA	27 (10.0)
PAD	56 (20.8)
VTE	18 (6.7)
Heart failure	47 (17.5)
CHA_2_DS_2_‐VASc score	3.8 ± 1.6
Drugs at enrollment	
Antiplatelet agents	159 (59.8)
Anticoagulant agents	7 (2.6)
Antiarrhythmics	17 (6.3)
Beta‐blockers	144 (54.1)
ACE inhibitors/ARBs	140 (52.6)
SGLT2 inhibitors	14 (5.3)
CCBs	89 (33.5)

*Note:* Values are expressed as *n* (%), mean ± standard deviation, or median [IQR].

Abbreviations: ACE = angiotensin‐converting enzyme inhibitors, ARBs = angiotensin receptor blockers, BMI = body mass index, CCBs = calcium channel blockers, CKD = chronic kidney disease;, COPD = chronic obstructive pulmonary disease, IQR = iInterquartile range, PAD = peripheral artery disease, SGLT2 = sodium‐glucose cotransporter‐2, SND = sinus node dysfunction;, TIA = transient ischemic attack, VTE = venous thromboembolism.

### 3.1 New‐Onset AHREs

During a median follow‐up of 28 months [20–36], 61 patients (22.7%) developed new‐onset AHREs (Figure 1). Patients with AHREs were older (80.5 ± 8.7 vs. 77.3 ± 11.7 years in those without AHREs; *p* = 0.047). Echocardiographic assessment revealed pre‐implant LAS reservoir of 18.7% [14.0–27.6] vs. 25.0% [17.0–34.2] (*p* = 0.003) and LAS contraction of 6.6% [3.6–10.0] vs. 13.3% [7.9–18.9] (*p* < 0.001) in patients with AHREs compared to those without. LAS conduit function did not differ between the two groups (12.4% [6.4–17.3] in patients with AHREs vs. 10.8% [6.7–16.5] in those without; *p* = 0.38). No difference between patients with and without AHREs was observed for LAVi, LVEF, LVEDV, LVESV, mitral E/A ratio, mean E/e' ratio, sPAP and TAPSE (Table 2).

**Table 2:** Baseline characteristics according to AHRE development after pacemaker implantation.

	No AHRE *N* = 208 (77.3%)	AHRE *N* = 61 (22.7%)	*p* value
Demographic and anthropometric characteristics			
Age (years)	77.3 ± 11.7	80.5 ± 8.7	**0.047**
BMI (Kg/m²)	25.7 [23.1–28.7]	25.7 [23.5–28.7]	0.42
Female gender	61 (29.3)	22 (36.1)	0.32
Cardiovascular risk factors and comorbidities			
Systemic hypertension	163 (78.4)	45 (73.8)	0.45
Diabetes mellitus	61 (29.3)	17 (27.9)	0.83
Smoking history	83 (39.9)	19 (31.1)	0.22
CKD	41 (19.7)	13 (21.3)	0.78
Dysthyroidism	21 (10.1)	10 (16.4)	0.18
COPD	17 (8.2)	4 (6.6)	0.68
History of stroke/TIA	24 (11.5)	3 (4.9)	0.13
PAD	44 (21.2)	12 (19.7)	0.80
VTE	13 (6.2)	5 (8.2)	0.59
Heart failure	36 (17.3)	11 (18.0)	0.90
CHA_2_DS_2_‐VASc score	3.8 ± 1.7	3.7 ± 1.5	0.71
Drugs at enrollment			
Antiplatelet agents	134 (61.5)	30 (48.4)	0.06
Anticoagulant agents	5 (2.4)	2 (3.4)	0.68
Antiarrhythmics	13 (6.2)	7 (11.5)	0.17
Beta clockers	115 (55.6)	29 (49.2)	0.38
ACE inhibitors/ARBs	110 (53.1)	30 (50.8)	0.76
SGLT2 inhibitors	12 (5.8)	2 (3.4)	0.47
CCBs	65 (31.4)	24 (40.7)	0.18
Indications for pacing			0.25
SND	43 (20.7)	11 (18.0)	
Second‐ or Third‐Degree AVB	134 (64.4)	35 (57.4)	
Reflex syncope	8 (3.8)	4 (6.6)	
Alternating Left and Right BBB	5 (2.4)	2 (3.3)	
Bifascicular/Trifascicular Block	8 (3.8)	7 (11.5)	
Multiple indications	10 (4.8)	2 (3.3)	
Pacing mode			0.65
DDD	171 (82.2)	47 (77.0)	
AAI	6 (2.9)	2 (3.3)	
VDD	31 (14.9)	12 (19.7)	
Pacing area			
Septum/Apex	118 (56.7)	34 (55.7)	0.60
LBB	85 (40.9)	24 (39.3)	
His bundle	5 (2.4)	3 (4.9)	
Echocardiography parameters			
MR	36 (17.3)	12 (19.7)	0.67
AS	11 (5.3)	7 (11.5)	0.09
TR	24 (11.5)	12 (19.7)	0.10
LVEDV (mL)	98.0 [79.0–121.0]	95.0 [79.0–129.0]	0.65
LVESV (mL)	40.0 [32.0–53.0]	40.0 [33.0–60.0]	0.53
LVEF (%)	57.0 [53.0–61.0]	56.0 [52.0–61.0]	0.57
LAVi (mL/m²)	28.0 [25.0–35.0]	32.0 [25.0–41.5]	0.11
Mitral E/A ratio	0.7 [0.5–0.9]	0.7 [0.5–1.0]	0.49
Mean E/e'ratio	8.0 [6.4–10.8]	8.0 [7.0–10.0]	0.62
sPAP (mmHg)	24.50 [15.00–30.0]	25.00 [15.00–35.0]	0.24
TAPSE (mm)	22.0 [20.0–25.0]	22.0 [20.0–25.0]	0.86
LAS reservoir (%)	25.0 [17.0– 34.2]	18.7 [14.0–27.6]	**0.003**
LAS contraction (%)	13.3[7.9–18.9]	6.6 [3.6–10.0]	**< 0.001**
LAS conduit (%)	10.8 [6.7–16.5]	12.4 [6.4–17.3]	0.38

*Note:* Values are expressed as *n* (%), mean ± standard deviation or median [IQR].

^a^Symptomatic Bifascicular/Trifascicular Block or Alternating Left and Right BBB.

Abbreviations: AAI = single lead atrial pacing, ACE = angiotensin‐converting enzyme inhibitors, AHRE = atrial high‐rate episodes, ARBs = angiotensin receptor blockers, AS = aortic stenosis, AVB = atrioventricular block, BBB = bundle branch block, BMI = body mass index, CCBs = calcium channel blockers, CKD = chronic kidney disease, COPD = chronic obstructive pulmonary disease, DDD = dual chamber atrioventricular pacing, IQR = interquartile range;,LAVi = left atrial volume Index, LAS = left atrial Strain; LBB = left bundle branch, LVEDV = left ventricular end‐diastolic Volume, LVEF = left ventricular ejection fraction, LVESV = left ventricular end‐systolic Volume, MR = mitral regurgitation, PAD = peripheral artery disease, PM = pacemaker, sPAP = systolic pulmonary artery pressure, SGLT2 = sodium‐glucose cotransporter‐2 Inhibitors, SND = sinus node dysfunction, TAPSE = tricuspid annular plane systolic excursion, TIA = transient ischemic attack, TR = tricuspid regurgitation, VDD = single lead atrio‐guided ventricularpacing, VTE = venous thromboembolism.

### 3.2 Predictive Value of LAS Parameters

**Figure 2:**
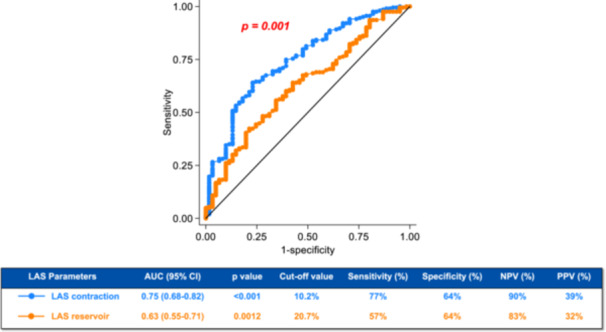
ROC curve analysis indicating the performance of LAS parameters for prediction of AHRE. AUC = area under the curve, LAS = left atrial strain, NPV = negative predictive value, PPV = positive predictive value, ROC = receiver‐operating characteristic.

ROC curve analysis (Figure 2) identified LAS contraction as superior to LAS reservoir for predicting AHREs [area under the curve (AUC) 0.75 [0.68–0.82] vs. 0.63 [0.55–0.71]; *p* < 0.001). LAS conduit was not a significant predictor of AHRE occurrence (AUC 0.54 [0.38–0.55]; *p* = 0.26). An optimal threshold for LAS contraction of 10.2% achieved a sensitivity of 77%, a specificity of 64%, a PPV of 39% and a NPV of 90% for AHRE occurrence. Stratification based on such cut‐off value of LAS contraction showed that patients with impaired LAS contraction (< 10.2%) had increased prevalence of chronic renal disease (25.8% vs. 15.4% in those with ≥ 10.2%; *p* = 0.034) and of tricuspid regurgitation (20.8% vs. 7.4%; *p* = 0.001), as well as a greater CHA₂DS₂‐VASc score (4.0 ± 1.7 vs. 3.6 ± 1.6; *p* = 0.05). Additionally, patients with LAS contraction < 10.2% exhibited increased LAVi (32.5 mL/m² [25.0–42.0] vs. 27.0 mL/m² [25.0–35.0] in those with preserved LAS contraction; *p* = 0.006), higher E/e′ ratio (9.8 [7.0–13.0] vs. 7.7 [6.0–9.5]; *p* < 0.001), and sPAP (25.0 mmHg [16.0–35.0] vs. 23.0 mmHg [15.0–28.0]; *p* = 0.006), along with lower TAPSE (21.0 mm [19.0–24.5] vs. 23.0 mm [20.0–25.0]; *p* = 0.009) (Table S3).

**Figure 3:**
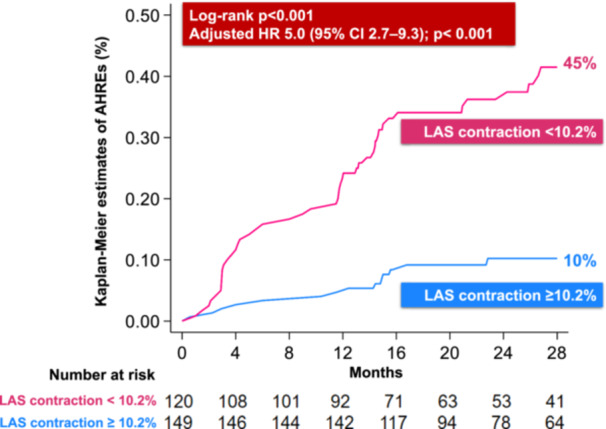
Kaplan–Meier curves for the incidence of AHRE during follow‐up in patients with preserved and reduced LAS contraction. AHREs = atrial high‐rate episodes, CI = confidence interval, LAS = left atrial strain; HR = hazard ratio.

**Figure 4:**
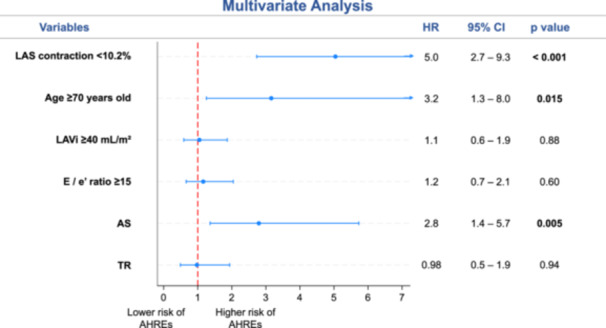
Multivariate analysis for the risk of AHRE during follow‐up. AHREs = atrial high‐rate episodes, AS = aortic stenosis, CI = confidence interval, CKD = chronic kidney disease, HR = hazard ratio, LAS = left atrial strain, MR = mitral regurgitation, TR = tricuspid rigurgitation.

Kaplan–Meier survival analysis (Figure 3) indicated that patients with LAS contraction < 10.2% had an increased cumulative incidence of AHREs (45%) during follow‐up vs those with preserved LAS contraction (10%; log‐rank *p* < 0.001). Multivariate analysis (Figure 4) identified that LAS contraction < 10.2% was the strongest predictor for AHRE development, with an adjusted HR of 5.0 (95% CI 2.7–9.3; *p* < 0.001). Other independent predictors were age ≥ 70 years (adjusted HR 3.2, 1.3–8.0; *p* = 0.015) and presence of aortic stenosis (adjusted HR 2.8, 1.4–5.7; *p* = 0.005).

**Figure 5:**
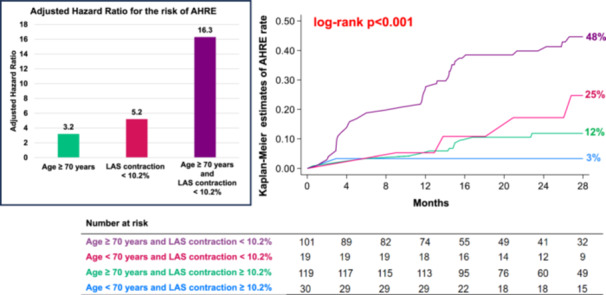
Left: Adjusted hazard ratios for AHREs in patients with age ≥ 70 years alone, with LAS contraction < 10.2% alone and with LAS contraction < 10.2% plus age ≥ 70 years (compared to patients with LAS contraction ≥ 10.2% and age < 70 years). Right: Estimated incidence of AHREs by Kaplan‐Meier method according to preoperative LAS contraction value and age. AHRE = atrial high‐rate episode, CI = confidence interval, LAS = left atrial strain, HR = hazard ratio.

A further risk stratification combining LAS contraction and age (Figure 5) demonstrated that patients with both LAS contraction < 10.2% and advanced age (≥ 70 years) had a 16‐fold higher risk of AHREs (adjusted HR 16.3, 2.0–131.7; *p* = 0.009), with a cumulative incidence of 48% during follow‐up. Patients with isolated impaired LAS contraction exhibited an intermediate cumulative incidence of 25%, whereas those with older age alone showed a 12% cumulative incidence and patients without these two conditions had the lowest risk of AHREs (3%) (log‐rank *p* < 0.001).

## 4 Discussion

This study represents to date the largest investigation evaluating the predictive value of LAS, particularly LAS contraction, for the occurrence of AHREs in patients with permanent PM implant.

Strain imaging has proven as a valuable tool to assess atrial dysfunction and remodeling, with well‐documented associations in various clinical settings [17, 18, 19]. LAS, derived from two‐dimensional speckle‐tracking echocardiography, is accurate in quantifying phasic LA function (reservoir, conduit, and contraction), each contributing to effective LV filling [22]. Among these, LAS reservoir reflects passive atrial compliance, LAS conduit captures passive emptying during early diastole, and LAS contraction quantifies the active booster pump atrial function in late diastole. A reduced LAS—especially in the reservoir and contraction phases—has been linked to elevated LV filling pressures, diastolic dysfunction, and adverse atrial remodeling, all conditions representing substrates for atrial arrhythmogenesis, including AF and AHREs [19, 20, 21]. Our analysis identified LAS contraction as the strongest independent predictor of future AHRE development in our population of PM recipients. In particular, patients with LAS contraction values ≤ 10.2% (e.g., a value representing a mild‐to‐moderate reduction in active atrial contraction) were significantly more likely to subsequently develop AHREs, with a fivefold increase. Notably, reduced LAS contraction not only may reflect an impaired atrial contractile mechanics, but also correlates with elevated LA pressures, LA interstitial fibrosis and myocyte degeneration, for example, hallmarks of atrial cardiomyopathy [23]. These pathophysiological changes can promote conduction heterogeneity, electrical remodeling and anisotropy, increasing the susceptibility to triggered activity and reentry, and ultimately predisposing patients to develop AHREs. According to our data, LAS conduit values were not associated with AHRE risk. Of note, while LAS reservoir was also independently related to an increased occurrence of AHREs, this relationship was less pronounced than LAS contraction. In addition, our analysis showed the association between reduced LAS contraction and more advanced cardiac remodeling, meaning larger left atria, impairment of right ventricular systolic function and worse left ventricular diastolic function.

Our findings reinforce and expand the results of the investigation by Wang et al., first reporting the predictive role of LAS contraction for AHREs in patients with CIEDs [20]. Of note, our study substantially differs from the Wang's study, as we included a larger and more homogeneous population, focusing on patients receiving a PM implant, and we used a more stringent AHRE definition following guideline‐recommended thresholds (> 5 min), rather than broader manufacturer‐specific criteria. Comparing the two papers, these distinctions may explain in our investigation the different LAS contraction threshold associated with increased AHRE occurrence versus what reported by Wang et al. (10.2% vs. 4.1%). Such 6% difference is clinically noteworthy and reliably evaluable, as this LAS contraction cutoff value was characterized in our study by high NPV (90%) and sensitivity (77%), but low specificity (64%), whereas by high specificity (88%) and low sensitivity (38%) in the Wang's investigation, where predictive values were not reported. Finally, we found a higher relative risk increase for AHREs in subjects with impaired LA contraction (fivefold vs. 20%). Indeed, it appears that Wang et al. focused on very high‐risk patients, while our study was aimed at assessing a “safety cut‐off” of LASct to better stratify before PM implant the risk of future AF episodes.

Indeed, while our protocol was not designed to assess pacing‐related effects, future research should explore whether preserving physiological activation patterns can mitigate LAS decline and its arrhythmogenic consequences [24]. Previous reports [25] indicating that cardiac resynchronization therapy can prevent new‐onset AF by diastole optimization are encouraging and, if replicated by CSP [8], may enforce the choice of these pacing modalities in patients with impaired pre‐implant LAS. Importantly, we also found that in patients having the combination of older age and impaired LA contraction there was a 16‐fold higher risk of AHREs, with approximately one half of patients developing these arrhythmic events during follow‐up. Overall, our findings emphasize the clinical importance of a comprehensive LAS assessment in patients undergoing PM implant, providing a foundation for improved risk stratification and individualized therapeutic strategies aimed at preventing AHREs and its associated complications.

### 4.1 Limitations

Our study is a retrospective, single‐center analysis, and this may limit external validity. The sample size, although larger than prior investigations, may still be underpowered to perform analyses in subgroups, such as in patients with different types of PM stimulation. Moreover, LAS was assessed just pre‐implantation, and this precluded the evaluation of how pacing might influence strain parameters over time. Finally, the relatively short follow‐up duration did not allow the assessment of clinical AF development or the risk of major cardiovascular events.

## 5 Conclusion

The impairment of LAS measures, especially of LA contraction, is an independent and robust predictor of AHREs in PM recipients. This underscores the importance of pre‐implantation assessment of atrial mechanics to identify patients at higher or lower risk for future atrial arrhythmias. Due to its high NPV, LAS evaluation is able to select low‐risk AF patients among elderly hypertensive patients, representing the majority of PM recipients, where unnecessary monitoring and overtreatment may be avoided. LAS evaluation may help clinicians to focus resources (especially extensive monitoring, costly high‐end devices) in those patients at higher risk. However, future longitudinal studies are needed to assess the interaction between pacing modalities, LAS parameters progression, biomarkers and arrhythmic risk, at the aim to develop a more personalized management framework useful in clinical practice.

## Conflicts of Interest

The authors declare no conflicts of interest.

## Supporting information

Suppl Table 1.

Suppl Table 2.

Suppl Table 3.

## Data Availability

The data that support the findings of this study are available from the corresponding author upon reasonable request.
