# Supplementary material for: Predictive Value of Left Atrial Strain for Atrial High‐Rate Episodes in Patients With Permanent Cardiac Pacing
Source: J Cardiovasc Electrophysiol. 2025 Nov 18;36(12):3343–52. doi: 10.1111/jce.70186 (PMC12697232; doi:10.1111/jce.70186)
Supplement: Supplementary file 1 — Suppl Table 1. [file JCE-36-3343-s001.docx]

**Supplementary Table 1.** Pacing indications and modalities.

|  | **Overall population**  **(n=269)** |
| --- | --- |
| *Indication for pacing* |  |
| SND | 54 (20.1) |
| Second- or third-degree AVB | 169 (62.8) |
| Reflex syncope | 12 (4.5) |
| Alternating Left and Right BBB | 7 (2.6) |
| Bifascicular/Trifascicular Block | 15 (5.6) |
| Multiple indications | 12 (4.5) |
| *Pacing mode* |  |
| DDD | 218 (81.0) |
| AAI | 8 (3.0) |
| VDD | 43 (16.0) |
| *Pacing area* |  |
| Septum/Apex | 152 (56.5) |
| LBBA | 109 (40.5) |
| His bundle | 8 (3.0) |

Values are expressed as n (%).

AAI = single lead Atrial pacing; AVB = Atrioventricular Block; BBB= Bundle Branch Block; DDD = Dual Chamber atrioventricular pacing; LBBA = Left Bundle Branch Area; SND = Sinus Node Dysfunction; VDD = single lead atrio-guided ventricular Pacing.
