# Supplementary material for: Predictive Value of Left Atrial Strain for Atrial High‐Rate Episodes in Patients With Permanent Cardiac Pacing
Source: J Cardiovasc Electrophysiol. 2025 Nov 18;36(12):3343–52. doi: 10.1111/jce.70186 (PMC12697232; doi:10.1111/jce.70186)
Supplement: Supplementary file 2 — Suppl Table 2. [file JCE-36-3343-s002.docx]

**Supplementary Table 2.** Pre-implant echocardiographic parameters in the overall population.

Values are expressed as n (%) or median [IQR].

|  | **Overall population**  **(n=269)** |
| --- | --- |
| Mild-to-moderate MR | 48 (17.8%) |
| Mild-to-moderate AS | 18 (6.7%) |
| Mild-to-moderate TR | 36 (13.4%) |
| LVEDV (mL) | 97.5 [79.0 - 122.0] |
| LVESV (mL) | 40.0 [32.0 - 55.0] |
| LVEF (%) | 57.0 [53.0 - 61.0] |
| LAVi (mL/m²) | 28.0 [25.0 - 38.0] |
| Mitral E/A ratio | 0.7 [0.5 - 0.9] |
| Mean E/e’ ratio | 8.0 [6.7 - 10.8] |
| sPAP (mmHg) | 25.0 [15.0 - 32.0] |
| TAPSE (mm) | 22.0 [20.0 - 25.0] |
| LAS reservoir (%) | 23.4 [16.4 - 32.4] |
| LAS contraction (%) | 10.7 [6.6 - 17.4] |
| LA conduit (%) | 11.4 [6.7 - 17.3] |

AS = Aortic stenosis; IQR=Interquartile Range; LAVi = Left Atrial Volume Index; LAS = Left Atrial Strain; LVEDV = Left Ventricular End-Diastolic Volume; LVEF = Left Ventricular Ejection Fraction; LVESV = Left Ventricular End-Systolic Volume; MR = Mitral regurgitation; sPAP = Systolic Pulmonary Artery Pressure; TAPSE = Tricuspid Annular Plane Systolic Excursion; TR = Tricuspid regurgitation.
