# Supplementary material for: Predictive Value of Left Atrial Strain for Atrial High‐Rate Episodes in Patients With Permanent Cardiac Pacing
Source: J Cardiovasc Electrophysiol. 2025 Nov 18;36(12):3343–52. doi: 10.1111/jce.70186 (PMC12697232; doi:10.1111/jce.70186)
Supplement: Supplementary file 3 — Suppl Table 3. [file JCE-36-3343-s003.doc]

**Supplementary Table 3**. Characteristics according to preserved or reduced LAS contraction before pacemaker implantation.

|  | LAS contraction ≥10.2% | LAS contraction <10.2% |  |
| --- | --- | --- | --- |
|  | N=149 (55.4) | 120 (44.6) | p value |
| *Demographic and anthropometric characteristics* | |  |  |
| Age (years) | 77.5±11.2 | 78.7±11.1 | 0.40 |
| BMI (Kg/m²) | 25.7 [23.4 - 28.7] | 25.4 [23.4 - 28.7] | 0.66 |
| Female gender | 36 (24.2) | 47 (39.2) | **0.008** |
| *Cardiovascular risk factors and comorbidities* | |  |  |
| Systemic hypertension | 118 (79.2%) | 90 (75.0%) | 0.41 |
| Diabetes mellitus | 41 (27.5) | 37 (30.8) | 0.55 |
| Smoking history | 59 (39.6) | 43 (35.8) | 0.53 |
| CKD | 23 (15.4) | 31 (25.8) | **0.034** |
| Dysthyroidism | 16 (10.7) | 15 (12.5) | 0.65 |
| COPD | 11 (7.4) | 10 (8.3) | 0.77 |
| History of Stroke/TIA | 15 (10.1) | 12 (10.0) | 0.99 |
| PAD | 30 (20.1) | 26 (21.7) | 0.76 |
| VTE | 12 (8.1) | 6 (5.0) | 0.32 |
| Heart Failure | 22 (14.8) | 25 (20.8) | 0.19 |
| CHA_2_DS_2_-VASc score | 3.6±1.6 | 4.0±1.7 | **0.05** |
| *Drugs at enrollment* |  |  |  |
| Antiplatelet agents | 85 (57.4) | 74 (62.7) | 0.38 |
| Anticoagulant agents | 6 (4.1) | 1 (0.8) | 0.11 |
| Antiarrhythmics | 13 (8.7) | 7 (5.8) | 0.37 |
| Beta blockers | 80 (54.1) | 64 (54.2) | 0.98 |
| ACE inhibitors / ARBs | 78 (52.7) | 62 (52.5) | 0.98 |
| SGLT2 inhibitors | 7 (4.7) | 7 (5.9) | 0.66 |
| CCBs | 53 (35.8) | 36 (30.5) | 0.36 |
| *Indications for pacing* |  |  | 0.06 |
| SND | 36 (24.2) | 18 (15.0) |  |
| Second- or Third-Degree AVB | 84 (56.4) | 85 (70.8) |  |
| Reflex syncope | 10 (6.7) | 2 (1.7) |  |
| Alternating Left and Right BBB | 5 (3.4) | 2 (1.7) |  |
| Bifascicular/Trifascicular Block | 9 (6.0) | 6 (5.0) |  |
| Multiple indications | 5 (3.4) | 7 (5.8) |  |
| *Pacing mode* |  |  | 0.26 |
| DDD | 125 (83.9) | 93 (77.5) |  |
| AAI | 5 (3.4) | 3 (2.5) |  |
| VDD | 19 (12.7) | 24 (20.0) |  |
| *Pacing area* |  |  | 0.77 |
| Septum/Apex | 87 (58.4) | 65 (54.2) |  |
| LBB | 58 (38.9) | 51 (42.5) |  |
| His bundle | 4 (2.7) | 4 (3.3) |  |
| *Echocardiography parameters* |  |  |  |
| MR | 21 (14.1) | 27 (22.5) | 0.07 |
| AS | 10 (6.7) | 8 (6.7) | 0.99 |
| TR | 11 (7.4) | 25 (20.8) | **0.001** |
| LVEDV (mL) | 98.0 [77.0 - 118.0] | 96.0 [82.0 - 129.0] | 0.14 |
| LVESV (mL) | 39.0 [32.0 - 52.0] | 42.0 [34.0 - 56.0] | 0.07 |
| LVEF (%) | 57.0 [53.0 - 62.0] | 57.0 [52.5 - 60.0] | 0.44 |
| LAVi (mL/m²) | 27.0 [25.0 - 35.0] | 32.5 [25.0 - 42.0] | **0.006** |
| Mitral E/A ratio | 0.7 [0.5 - 0.9] | 0.7 [0.5 - 1.0] | 0.63 |
| Mean E/e’ ratio | 7.7 [6.0 - 9.5] | 9.8 [7.0 - 13.0] | **<0.001** |
| sPAP (mmHg) | 23.0 [15.0 - 28.0] | 25.0 [16.0 - 35.0] | **0.006** |
| TAPSE (mm) | 23.0 [20.0 - 25.0] | 21.0 [19.0 - 24.5] | **0.009** |
| LAS reservoir (%) | 29.5 [23.2 - 36.9] | 17.0 [11.8 - 21.7] | **<0.001** |
| LAS conduit (%) | 11.9 [7. 5 - 17.7] | 10.2 [5.7 - 16.3] | 0.13 |

Values are expressed as n (%), mean ± standard deviation or median [IQR].

*Symptomatic Bifascicular/Trifascicular Block or Alternating Left and Right BBB.

AAI = single lead Atrial Pacing; ACE = Angiotensin-Converting Enzyme Inhibitors; AHRE= Atrial high-rate episodes; ARBs = Angiotensin Receptor Blockers; AS = Aortic Stenosis; AVB = Atrioventricular Block; BBB= Bundle Branch Block; BMI = Body Mass Index; CCBs = Calcium Channel Blockers; CKD = Chronic Kidney Disease; COPD = Chronic Obstructive Pulmonary Disease; DDD = Dual chamber atrioventricular pacing; IQR = Interquartile Range; LAVi = Left Atrial Volume Index; LAS = Left Atrial Strain; LVEDV = Left Ventricular End-Diastolic Volume; LVEF = Left Ventricular Ejection Fraction; LVESV = Left Ventricular End-Systolic Volume; MR = Mitral Regurgitation; PAD = Peripheral Artery Disease; PM = Pacemaker; sPAP = Systolic Pulmonary Artery Pressure; SGLT2 = Sodium-Glucose Cotransporter-2 Inhibitors; SND = Sinus Node Dysfunction; TAPSE = Tricuspid Annular Plane Systolic Excursion; TIA = Transient Ischemic Attack; TR = Tricuspid Regurgitation; VDD = single lead atrio-guided ventricular Pacing; VTE = Venous Thromboembolism.
